# Health‐related quality of life and related factors among a sample of older people with cognitive impairment

**DOI:** 10.1002/nop2.265

**Published:** 2019-03-19

**Authors:** Line Christiansen, Johan Sanmartin Berglund, Catharina Lindberg, Peter Anderberg, Lisa Skär

**Affiliations:** ^1^ Department of Health Blekinge Institute of Technology Karlskrona Sweden

**Keywords:** ageing, cognitive impairment, EuroQol, health‐related quality of life, Short‐Form Health Survey‐12

## Abstract

**Aim:**

This study aimed to identify factors affecting health‐related quality of life (HRQoL) of older adults with cognitive impairment and to describe the association of these factors with different components of HRQoL.

**Design:**

A cross‐sectional, descriptive research design was used.

**Methods:**

Data were collected from 247 individuals aged 60 years and older from a Swedish longitudinal cohort study. The Short‐Form Health Survey‐12 (SF‐12) and EuroQol (EQ‐5D) were used to assess HRQoL. The data were analysed using descriptive and comparative statistics.

**Results:**

The present study identified several factors that influenced HRQoL of older adults with cognitive impairment. The results of a multiple logistic regression analysis revealed that the following factors were associated with physical and mental HRQoL: dependency in activities of daily living (ADL), receiving informal care and feelings of loneliness and pain.

## 1 INTRODUCTION

In an ageing population, the risk of poor health and cognitive impairment increases with age (Teeters et al., 2016). According to Murman (2015), cognition is critical for functional independence as people age. Although many older adults retain good health in old age, cognitive impairment can lead to increased dependency in daily life (Ahn et al., 2009; Reppermund et al., 2013). Such dependency can affect different aspects of health and overall quality of life (QoL) (Ahn et al., 2009; Reppermund et al., 2013). To support health and maintain independent living in old age, it is important to understand the challenges that older individuals face, including the most important factors that influence health and QoL. There is growing interest in measuring these challenges and the factors affecting health‐related quality of life (HRQoL) as outcome measures in the field of health research (Leow et al., 2013).

## 2 BACKGROUND

There are several definitions of the concept of health (Bircher, 2005; Diener, 2000; Guyatt, 1993; Saracci, 1997), including the widely used definition by the World Health Organization (WHO, 1948). According to its definition, health is “a state of complete physical, mental and social well‐being and not merely the absence of disease and infirmity” (p. 100). Despite the many definitions, a measure of health status is difficult to establish using a single measure. Mainly because health varies greatly among individuals and is influenced both by culture and a person's age (Hernandez, Blazer & Institute of Medicine (US) Committee on Assessing Interactions Among Social, Behavioral, and Genetic Factors in Health 2006). But also, because health comprises many interrelated components, such as physical functioning, cognitive functioning, mental well‐being and self‐regulation (Leist, Kulmala, & Nyqvist, 2014). Furthermore, health is often described as a positive concept that has been operationalized in terms of self‐reported (mental and physical) health status and broader health status, which includes the ability to continue with daily social role functioning (Huber et al., 2011; Singh & Dixit, 2010). Thus, the establishment of health status should be based on definitions of the concept of health that are multidimensional (Bowling, 2017).

Ageing is associated with both biological and psychological changes, including cognitive impairment, such as dementia (Glisky, 2007). Dementia is a multifactorial disorder, which is characterized by progressive deterioration in memory and other cognitive domains (Winblad et al., 2016). Both the prevalence and incidence of dementia increase significantly with advancing age, and it is a major cause of functional dependence, institutionalization, poor QoL and mortality in older adults (Prince et al., 2015; Qiu, von Strauss, Bäckman, Winblad, & Fratiglioni, 2013). The negative impacts of dementia on health and QoL of older adults are well established (Cooper et al., 2012). The effects of dementia on QoL encompass cognitive function, activities of daily living (ADL), social interactions and mental health (Beerens et al., 2014). Previous research demonstrates that even early stages of dementia (i.e. mild dementia or mild cognitive impairment) adversely affect QoL (Bárrios et al., 2013). Research also found a strong association between health and QoL among older adults (Baernholdt, Hinton, Yan, Rose, & Mattos, 2012). Furthermore, self‐rated health is shown to be strongly associated with subjective views of QoL in people with dementia (Orgeta, Edwards, Hounsome, Orrell, & Woods, 2015).

Despite the aforementioned findings, the relationship between health and QoL is complex (Borglin, 2005). To measure the health status of older adults with cognitive impairment, their health needs to be based on a definition that encompasses QoL. The application of the concept of QoL in the area of health and illness is referred to as HRQoL. HRQoL is a multidimensional concept that has evolved since the 1980s to encompass components of overall QoL that can be clearly shown to affect health (Bowling, 2001). These components include physical, mental, emotional and social functioning (Bowling, 2001). HRQoL is often used in scientific research to measure health status and population health outcomes since it provides a broad summary measure of perceived health (Kindig, Asada, & Booske, 2008). Previous research of older populations shows that the negative impact of cognitive dysfunction on HRQoL increased with the severity of cognitive dysfunction and is associated with a lower physical and mental health (Pan et al., 2015). In addition, poor mental health is associated with cognitive impairment in old age (Bauermeister & Bunce, 2015).

Many older adults with cognitive decline can continue to live at home in the early stages of the disorder, with support from a family member or relative, who usually serves as an informal caregiver (Lethin, 2016). However, as the condition progresses, the individual may suffer a loss of independence and have an increased need for care assistance with both basic ADL, such as functional mobility, dressing, bathing and toileting and instrumental activities of daily living (IADL) (e.g. preparing meals, cleaning, cooking and handling finances) (DeFries, McGuire, Andresen, Brumback, & Anderson, 2009; Schulz & Martire, 2004). A study of functional ability in older adults demonstrated that individuals with mild cognitive impairment had poorer IADL functioning as compared with that of cognitively intact older individuals (Johansson, Marcusson, & Wressle, ). The same study found that requiring help with ADL had a significant negative effect on older adults HRQoL. Therefore, measuring HRQoL has become an important objective in dementia research, where the importance of valuing the perspective of the person with dementia is emphasized (Hounsome, Orrell, & Edwards, 2011). There has been a recent increase in studies investigating the relationship between cognition and HRQoL. However, research about factors of significance for the HRQoL among this group of older adults is still sparse. Hence, the purpose of this study was to identify factors affecting HRQoL among older adults with cognitive impairment and to discuss the association of these factors in relation to different components of HRQoL.

## 3 METHODS

### 3.1 Design

A cross‐sectional, descriptive research design was chosen. The Short‐Form Health Survey‐12 (SF‐12) and EuroQol (EQ‐5D) were used to assess HRQoL among older adults with cognitive impairment. The data were analysed using descriptive and comparative statistics.

### 3.2 Study population

The study population consisted of individuals who took part in the Swedish National Study on Aging and Care (SNAC). SNAC is a longitudinal, multicentre, cohort study, which commenced in the county of Blekinge, Sweden in 2001–2003 (Lagergren et al., 2004). The original study consisted of 1,402 participants aged 60–96 years. All the participants underwent clinical examinations, focusing on different aspects of ageing. The participants were followed up every third or sixth year, depending on their age, until dropout. The present study is based on participants (*N = *247) selected from the SNAC cohort study in 2007–2009, who scored 20–26 points in the mini‐mental state examination test (MMSE) which was used as an inclusion criteria for this study. The MMSE contains questions targeting memory, learning and orientation, with scores from 0–30 points, where a score of less than 26 points or less indicates cognitive difficulties (i.e. mild cognitive impairment or mild dementia) (Folstein, Folstein, & McHugh, 1975).

### 3.3 Measures

The outcome variable in this study was HRQoL. The study employed Ware's (1987) multidimensional perspective of HRQoL, where there are five inherent health concepts: physical health, mental health, social functioning, role functioning and general well‐being. In Ware's definition of HRQoL, the goal of health care is to maximize the health components of QoL. In this study, we applied this definition in the application of two health measurement scales: the SF‐12 and EuroQol (EQ‐5D). Since both scales aim to measure functioning, well‐being and general health status they are considered appropriate to answer the purpose of this study.

With regard to the measure of HRQoL and the health measurement scales used in this study, there were some internal dropouts. A comparison of the study population that completed SF‐12 (*N = *179) and the population that did not complete SF‐12 (*N = *68) showed that the non‐participants/non‐responders belonged to the oldest participants (81–87 and 90–105 years). In contrast, a comparison of participants who completed the EQ‐5D (*N = *237) and those that did not (*N = *10) revealed that most the non‐participants/non‐responders belonged to the youngest participants (60–66 years). Thus, the final study consisted of 247 participants who were included in the health measurements analysis.

### 3.4 Assessment of HRQoL

#### 3.4.1 The Short‐Form Health Survey‐12

The SF‐12 is a generic, standardized questionnaire used to measure two components of HRQoL: physical and mental health. It is a shorter, yet valid alternative to the most widely used health status scale: the Short‐Form‐36 Health Survey Questionnaire (SF‐36). The SF‐12 reproduces the physical and mental component summary scores (PCS/MCS) of the SF‐36. As it has a broad generic approach and does not target a specific age or disease group, it is suitable for use with larger samples where broader health outcomes are monitored (Ware, Kosinski, & Keller, 1996). In addition, as the SF‐12 contains fewer questions and takes less time to complete than the SF‐36, it is more appropriate for use with older adults with common geriatric diseases, such as dementia (Jenkinson & Layte, 1997; Ware et al., 1996). The SF‐12 is weighted and summed to provide easily interpretable scales for the two health components.

In this study, the results are presented as PCS‐12 and MCS‐12, where scores range from zero (lowest HRQoL)–100 (highest HRQoL). In a previous study of the general Swedish population aged 75 years and older, scores of 40.3 (*SD* 11.6) and 51.5 (*SD* 11.0) on the PCS‐12 and MCS‐12, respectively, were considered normal (Sullivan, Karlsson, & Taft, 1997). In this study, a cut‐point on the 25th percentile was used to establish the limit for low and high HRQoL since no validated cut‐points calculated for an older adult population with cognitive impairment was described in the literature. When establishing cut‐points from a continuous scale, distribution‐based or anchor‐based strategies are usually used in the area of health measurement (Streiner, 2014). The present study applied a distribution‐based strategy. A value of 27 or less denoted low physical HRQoL, and a value of 46 or less denoted low mental HRQoL.

#### 3.4.2 The EuroQoL

The EQ‐5D is a generic, multidimensional health self‐report questionnaire, designed to generate a single index value for each health state (Bowling, 2017). The questionnaire contains five health‐related items: mobility, self‐care, usual activities, pain/discomfort and anxiety/depression. The respondents classify their own health status in three levels per item. Utility scores quantify HRQoL along a continuum that ranges from −0.59 (worst health) –1.00 (perfect health). Respondents are also asked to rate their current health status on a 100‐point visual analogue scale (VAS), where higher points represent the best imaginable health state (Bowling, 2017).

The analysis of the EQ‐5D index is based on a time trade‐off (TTO) value set and the VAS. As there is no Swedish TTO value set for EQ‐5D health states, the UK EQ‐5D index tariff was used in this study to obtain values for health status (Dolan, 1996). A cut‐off on the 25th percentile was used to establish the limit for low and high HRQoL. Cronbach's alpha for the index was calculated as 0.692.

### 3.5 Assessment of dependency in functional ability

The ADL and IADL indices developed by Katz, Ford, Moskowitz, Jackson, and Jaffe (1963) and Lawton and Brody (1969), respectively, were used to assess the participant's dependency in functional ability in everyday life. The ADL index describes the functional status of elderly and chronically ill patients. It assesses independence in basic ADL, such as personal hygiene care, toileting and dressing. The index is composed of 6 items, where the individual is graded on an ordinal 3‐point scale, with lower scores indicating full functioning (Bowling, 2017). In this study, each item was rated dichotomously (0 = dependent and 1 = independent) and then summarized to create an index. The same procedure was performed for the IADL scale, which is composed of eight items. On the IADL scale, higher scores indicate greater functional ability and the scores are corrected according to the gender of the participant.

### 3.6 Statistical analysis

All statistical analyses were performed using the Statistical Package for Social Science, version 24.0 (SPSS Inc., NY, USA). The clinical and socio‐demographic characteristics of the study sample were analysed using descriptive and comparative statistics. The results are presented as absolute frequency (*N*) and relative frequency (%). As the data were not distributed normally in non‐parametric tests, the Mann–Whitney *U* test and Kruskal–Wallis test were conducted for comparisons between sex and age. Univariate analyses (i.e. correlations) with Spearman's rho (*r*_s_) and binary logistic regression analysis were performed to assess the importance of independent variables. Furthermore, a multiple logistic regression analysis was performed to determine the relation between the outcome variables (physical and mental HRQoL) and independent variables. When conducting the regression models, all the independent variables were dichotomized and backward (likelihood ratio) was used when adjusting for sex, age and education. In model comparisons, the likelihood ratio test and goodness‐of‐fit test of Hosmer and Lemeshow (2013) were used to determine how well the observed data corresponded to the predicted data in the models. The results obtained in the multiple logistic regression analysis are presented as odds ratios (ORs), with their 95% confidence intervals (CIs) and *p*‐values for statistical significance (*p < *0.05) (Altman, 1991).

### 3.7 Ethical consideration

This study was conducted in accordance with the ethical principles for research outlined in the Declaration of Helsinki (WMA, 2013). All the participants provided written informed consent before entering the SNAC study, and Research Ethics Committee approval was obtained from the regional ethics review board in Lund (LU No. 650‐00 and No. 744‐00).

## 4 RESULTS

### 4.1 Characteristics

The distribution according to sex in the present study (*N = *247) was 57.9% (*N = *143) females and 42.1% (*N = *104) males. The mean age of the female participants was 82.9 years (*SD*: 9.4), whereas the mean age of the male participants was 81.6 years (*SD*: 8.4). Most of the participants were married 51.2% (*N = *87) or widowed 44.5% (*N = *94). The remainder were unmarried 5.2% (*N = *11) or divorced 9% (*N = *19). Most of the study population was composed of pensioners 89% (*N = *202), and 29.4% (*N = *67) had an education level higher than elementary school.

Regarding self‐rated physical well‐being and cognitive function, most (55.2%; *N = *107) of the study population reported moderate health and 53.8% (*N = *133) reported mild complaints in memory, where women accounted for 54.2% (*N = *77). Concerning the incidence of pain, most of the participants 65.6% (*N = *127) reported having moderate pain, where more women 69.2% (*N = *74) than men reported pain. Furthermore, 8.7% (*N = *18) of the participants reported living in institutional care while 91.3% (*N = *189) were living in community dwelling. Among those who were living in community dwelling, 59.3% (*N = *127) were living alone. In addition, significantly more women than men (77% vs. 35.9%, *p *< 0.01) were living alone. Overall, 48% (*N = *97) of the study population reported that they received informal care. The characteristics of the study sample are presented in Table 1.

**Table 1:** Study population characteristics (*N* = 247)

Variable	Male *N* (%)	Female *N* (%)	Total *N* (%)	*p‐*value*
Age groups (*N* = 247)				
60–66	10 (9.6)	16 (11.2)	26 (10.5)	0.282
72–78	28 (26.9)	24 (16.8)	52 (21.1)	
81–87	40 (38.5)	60 (42.0)	100 (40.5)	
90–105	26 (25.0)	43 (30.1)	69 (27.9)	
Marital status (*N* = 211)				
Married	57 (61.3)	30 (25.4)	87 (41.2)	<0.001
Widow	24 (25.8)	70 (59.3)	94 (44.5)	
Unmarried	7 (7.5)	4 (3.4)	11 (5.2)	
Divorced	5 (5.4)	14 (11.9)	19 (9.0)	
Living status (*N* = 214)				
Alone	33 (35.1)	94 (75.8)	127 (59.3)	<0.001
Together	59 (62.8)	28 (22.6)	87 (39.9)	
Receive informal care	30 (36.1)	67 (56.3)	97 (48.0)	
Do not receive informal care	53 (63.9)	52 (43.7)	105 (52.0)	
Housing (*N* = 207)				
Community dwelling	86 (93.5)	103 (89.6)	189 (91.3)	0.457
Residential care facility	6 (6.5)	12 (10.4)	18 (8.7)	
Education (*N* = 228)				
Elementary school or equivalent	69 (69.7)	92 (71.3)	161 (70.6)	0.884
Higher than elementary school	30 (30.3)	37 (28.7)	67 (29.4)	
Occupation (*N* = 227)				
Acquisition workers	10 (10.5)	15 (11.4)	25 (11.0)	1.000
Pensioner	85 (89.5)	117 (88.6)	202 (89.0)	
Financial status (*N* = 186)				
High	74 (85.1)	76 (71.0)	150 (77.3)	0.060
Low	10 (11.5)	26 (24.3)	36 (18.6)	
Cognitive function (*N* = 246)				
Self‐rated memory				
No complaints	20 (19.2)	38 (26.6)	58 (23.5)	0.246
Mild complaints	56 (53.8)	77 (54.2)	133 (53.8)	
Severe complaints	28 (26.9)	27 (18.9)	55 (22.3)	
Activities of daily living (*N* = 247)				
Dependent	8 (7.7)	17 (11.9)	25 (10.1)	0.297
Independent	96 (92.3)	126 (88.1)	222 (89.9)	
Instrumental activities of daily living (*N* = 235)				
Dependent	23 (22.8)	28 (20.9)	51 (21.7)	0.751
Independent	78 (77.2)	106 (79.1)	184 (78.3)	
Physical well‐being (*N* = 188)				
Self‐rated health				
Good health	32 (36.8)	34 (31.8)	66 (34.0)	0.727
Moderate health	45 (51.7)	62 (57.9)	107 (55.2)	
Bad health	9 (10.3)	6 (5.6)	15 (7.7)	
Pain (*N* = 191)				
No pain	29 (33.3)	25 (23.4)	54 (28.3)	0.525
Moderate pain	53 (60.9)	74 (69.2)	127 (65.6)	
Severe pain	4 (4.6)	6 (5.6)	10 (5.2)	

Note

The Mann–Whitney *U* test was used in the comparisons between sex.

* *p* < 0.05 significance level for the difference between sex.

### 4.2 The SF‐12, Physical component summary scores and Mental component summary scores

As shown by the analysis of the two health measures (physical health and mental health), an almost equal proportion of women and men had a low Physical component summary scores (PCS) (23.2% vs. 23.8%) and low Mental component summary scores (MCS) (34.7% vs. 32.1%). Evaluation of PCS according to sex showed that the mean PCS of both males and females (40.0 vs. 37.6) was close to the reference value (40.3) of a general Swedish geriatric population. Similar results were observed for the evaluation of MCS, where the mean MCS of males and females (52.2 vs. 51.7) was close to the reference value (51.5). However, there were significant differences (*p *= 0.023) in the PCS of the participants in the different age groups (Table 2).

**Table 2:** HRQoL outcome for gender and age cohorts

Outcome	Variable	*N*	Mean (*SD*)	Ref.value^a^	*p*‐value*
PCS	Male	84	40.0 (12.3)	40.3	0.217
	Female	95	37.6 (12.0)		
	60–66	24	44.5 (12.2)		0.023
	72–78	44	40.1 (11.7)		
	81–87	74	39.3 (11.7)		
	90–105	37	31.3 (10.7)		
MCS	Male	84	52.2 (9.7)	51.5	0.729
	Female	95	51.7 (9.1)		
	60–66	24	53.1 (9.3)		0.301
	72–78	44	52.5 (8.5)		
	81–87	74	52.2 (9.0)		
	90–105	37	49.5 (11.0)		

Note

HRQoL outcome is based on physical component summary (PCS) and mental component summary (MCS).

a PCS and MCS reference values for the general Swedish geriatric population (Sullivan et al., 1997).

* *p* < 0.05 significance level for the difference between sex and age.

### 4.3 The EQ‐5D scores

According to the EQ‐5D, an almost equal proportion of women and men had a low HRQoL (18.5% vs. 19.6%), with no statistically significant difference (*p *= 0.730). Concerning the different components of HRQoL, there were no sex‐related differences in any of the EQ‐5D components (Table 3). When the data were compared according to age groups, the results showed significant differences in the EQ‐5D components' *mobility* (*p *< 0.01), *self‐care* (*p *= 0.013) and *usual activities* (*p *= 0.006) between the different age groups (Table 4).

**Table 3:** Distribution of responses of the EQ‐5D dimensions by gender

Dimension	Respond	Male *N* (%)	Female *N* (%)	Total *N* (%)	*p‐*value*
Mobility (*N* = 190)	No problems	49 (57.0)	50 (48.1)	99 (52.1)	0.811
	Some problems	37 (43.0)	53 (51.0)	90 (47.4)	
	Extreme problems	–	1 (1.0)	1 (0.5)	
Self‐care (*N* = 191)	No problems	75 (87.2)	94 (89.5)	169 (88.5)	0.285
	Some problems	9 (10.5)	8 (7.6)	17 (8.9)	
	Extreme problems	2 (2.3)	3 (2.9)	5 (2.6)	
Usual activities (*N* = 189)	No problems	67 (78.8)	84 (80.8)	151 (79.9)	0.848
	Some problems	16 (18.8)	15 (14.4)	31 (16.4)	
	Extreme problems	2 (2.4)	5 (4.8)	7 (3.7)	
Pain/discomfort (*N* = 191)	No problems	29 (33.7)	25 (23.8)	54 (28.3)	0.721
	Some problems	53 (61.6)	74 (70.5)	127 (66.5)	
	Extreme problems	4 (4.7)	6 (5.7)	10 (5.2)	
Anxiety/depression (*N* = 190)	No problems	62 (72.1)	56 (53.8)	118 (62.1)	0.279
	Some problems	24 (27.9)	46 (44.2)	70 (36.8)	
	Extreme problems		2 (1.9)	2 (1.1)	

Note

The Mann–Whitney *U* test was used in the comparisons between sex.

* *p* < 0.05 significance level for the difference between sex.

**Table 4:** Distribution of responses of the EQ‐5D dimensions by age

Dimension	Respond	60–66 years *N* (%)	72–78 years *N* (%)	81–87 years *N* (%)	90–105 years *N* (%)	*p*–value*
Mobility (*N* = 190)	No problems	19 (79.2)	28 (63.6)	47 (57.3)	5 (12.5)	<0.01
	Some problems	5 (20.8)	16 (36.4)	34 (41.5)	35 (87.5)	
	Extreme problems	–	–	1 (1.2)	–	
Self‐care (*N* = 191)	No problems	23 (95.8)	42 (95.5)	74 (89.2)	30 (75.0)	0.013
	Some problems	1 (4.2)	2 (4.5)	6 (7.2)	8 (20.0)	
	Extreme problems	–	–	3 (3.6)	2 (5.0)	
Usual activities (*N* = 189)	No problems	22 (91.7)	39 (88.6)	66 (80.5)	24 (61.5)	0.006
	Some problems	1 (4.2)	5 (11.4)	13 (15.9)	12 (30.8)	
	Extreme problems	1 (4.2)	–	3 (3.7)	3 (7.7)	
Pain/discomfort (*N* = 191)	No problems	10(41.7)	11 (25.0)	21 (25.3)	12 (30.5)	0.478
	Some problems	13 (54.2)	31 (70.5)	57 (68.7)	26 (65.0)	
	Extreme problems	1 (4.2)	2 (4.5)	5 (6.0)	2 (5.0)	
Anxiety/depression (*N* = 190)	No problems	14 (60.9)	30 (68.2)	52 (32.7)	22 (55.0)	0.572
	Some problems	9 (39.1)	14 (31.8)	31 (37.3)	16 (40.0)	
	Extreme problems	–	–	–	2 (5.0)	

Note

The Kruskal–Wallis test was used for comparisons between age groups.

* *p* < 0.05 significance level for the difference between age groups.

### 4.4 Dependency in functional ability

When the ability to perform ADL was analysed, the results showed that 89.9% (*N = *222) of the participants had full functioning, 6.4% (*N = *16) had moderate functioning and 3.6% (*N = *9) had severe functional impairment. Regarding the ability to perform ADL, there were no significant sex‐related differences in dependency in functional ability (*p *= 0.297), although more women than men (11.9% vs. 7.7%) were dependent on support to accomplish ADL (Table 1). In two ADL functions (i.e. *bathing* and *continence*)*,* there were significant differences between women and men (*p *= 0.036 and *p *= 0.009*,*respectively). In total, 86.6% (*N = *214) of the participants were able to independently perform bathing, whereas 13.4% (*N = *33) of the population was dependent on support. Concerning the ADL function “continence,” 72.9% (*N = *180) of the participants were able to independently exercise complete self‐control over urination and defecation, whereas 27.1% (*N = *67) of the participants were partially or totally dependent on support. In both cases, women were more dependent than men.

Regarding the ability to perform IADL, the comparison between the means for women and men revealed no significant difference in overall functional ability (*p *= 0.731), although more men than women were dependent on support to accomplish IADL (22.8% vs. 20.9%) (Table 1). There was a significant difference between women and men in one IADL function: *mode of transportation* (*p *= 0.008). Women were less likely than men to travel independently, either in their own car or on public transportation and they were less likely than men to travel on their own (38.7% vs. 22.1%).

### 4.5 The relation between HRQoL and functional ability by gender

The mean HRQoL score of women and men was similar, both in the EQ‐5D (19% vs. 20%) and SF‐12 (23% vs. 24%; PCS‐12) (35% vs. 32%; MCS‐12). In terms of the relation between HRQoL and functional ability, the results of the univariate analysis revealed a weak correlation between physical HRQoL (PCS‐12), ADL and IADL (*r*_s_ = 0.309–0.330, *p *< 0.01), indicating that dependency in functional ability was associated with lower HRQoL. According to the results of the EQ‐5D, participants who independently performed ADL (91.7%) had a statistically significant higher HRQoL (*p *= 0.031) than those who were dependent on support to accomplish ADL (8.3%). Among men and women who were dependent in ADL, women (10.9%) had a higher HRQoL than men (4.9%), as shown by the EQ‐5D. This was also the case for physical HRQoL (PCS‐12) (5.5% vs. 0%) and mental HRQoL (MCS‐12) (9.7% vs. 5.3%), although the difference was not statistically significant (*p *= 0.626; EQ‐5D; *p *= 0.251; PCS‐12; *p = *1.000; MCS‐12). There were significant sex‐related differences in the relationship between physical HRQoL (PCS‐12) and functional ability in ADL (*p *= 0.009 for women; *p *= 0.003 for men) and IADL (*p *= 0.012 for women; *p *= 0.007 for men).

The multiple logistic regression analysis resulted in two models including the outcome variables physical HRQoL (PCS‐12) and mental HRQoL (MCS‐12). The first model (Table 5A) revealed that low physical HRQoL was associated with receiving informal care (OR: 10.373), dependency in ADL (OR: 14.891) and experiencing moderate to severe pain (OR: 5.273). The second model (Table 5B) revealed an association between mental HRQoL and loneliness (OR: 3.829). Participants who sometimes experienced loneliness were more likely to have a low mental HRQoL than those who never felt lonely. More women than men reported that they sometimes felt lonely (52.4% vs. 43%, *p *= 0.244). In addition, there was a weak correlation between loneliness and living alone (*r*_s_ = 0.390, *p *< 0.01).

**Table 5:** (A) Logistic regression analysis (backward: LR) of factors associated with physical HRQoL. (B) Logistic regression analysis (backward: LR) of factors associated with mental HRQoL

(A)			
Coefficient	Odds ratio (e^b^)	95% C.I. for OR	*p‐*value*
Informal care			
Receive	10.373		
Do not receive	(Ref)	3.373–31.900	0.000
ADL			
Dependent	14.891		
Independent	(Ref)	2.518–88.057	0.003
Pain			
Moderate/severe	5.273		
No pain	(Ref)	1.097–25.353	0.038

(B)			
Coefficient	Odds ratio (e^b^)	95% C.I. for OR	*p‐*value*
Loneliness			
Lonely	3.829	1.630–8.991	0.002
Not lonely	(Ref)		

Note

(A) HRQoL: health‐related quality of life derived from the Physical Health Composite Scale (PCS‐12) is dichotomized (low HRQoL = 1 as reference). Cox and Snell *R* ^2^ = 0.298. Nagelkerke's *R* ^2^ = 0.439. *N* = 179. (B) HRQoL: health‐related quality of life derived from the Mental Health Composite Scale (MCS‐12) is dichotomized (low HRQoL = 1 as reference). Cox and Snell *R* ^2^ = 0.158. Nagelkerke's *R* ^2^ = 0.223. *N* = 179.

* *p* < 0.05 significance level.

## 5 DISCUSSION

The present study aimed to identify and describe factors associated with HRQoL among older adults with cognitive impairment. The results revealed that HRQoL of the study population as regards physical and mental health was similar to that of the general Swedish geriatric population (Sullivan et al., 1997), despite having cognitive impairment. In addition, there were hardly any differences in HRQoL between males and females. Earlier research (Franzén, Saveman, & Blomqvist, 2007; Hopman et al., 2009) that studied HRQoL in older adults with chronic diseases showed that chronic heart failure was strongly associated with reduced HRQoL. Further, comorbid conditions, such as diabetes and respiratory diseases, as well as female sex and older age, were associated with poorer physical health.

The results further revealed an association between pain and functional ability in relation to physical health. Having moderate to severe pain or being dependent in ADL was correlated with lower physical health. This is in line with previous research (Fagerström & Borglin, 2013), which showed that functional ability in older adults was associated with both physical and mental health. In the present study, the dependency levels of women and men differed, with women in general more dependent than men on support to perform daily activities. Despite this finding, the results indicated that women who were dependent on support to accomplish ADL had a higher HRQoL than men who were dependent, both in terms of physical and mental health. Previous studies of the functional ability of older adults with cognitive impairment did not clarify the impact of basic ADL functioning on HRQoL (Mlinac & Feng, 2016; Pusswald et al., 2015). According to Bowling (2017), most measures of functioning focused narrowly on mobility, self‐care and instrumental tasks and did not always include assessments of more meaningful aspects of a person's functioning (e.g. the time taken to complete a task). Many measures of functional ability have been developed that are suited to a particular age or disease group and validated with people living in institutions or in the community (Comans et al., 2018; Wilson et al., 2011). Combining such measurements with self‐rated health measurements, as was done in the present study, may provide a clearer picture of a person's overall functional status. Knowledge about functional status could thereby support HRQoL.

The results of the current study also identified an association between loneliness and mental health. Participants who sometimes experienced loneliness were more likely to have a low mental HRQoL than those who never felt lonely. According to previous research, loneliness and social isolation were associated with mental illness in old age (Loboprabhu & Molinari, 2012; Wenger, Davies, Shahtahmasebi, & Scott, 1996; Wilkes, 1978). Studies also found that the absence of loneliness was related to a good QoL among older adults (Gerino, Rollè, Sechi, & Brustia, 2017; Kang, Park, and Wallace (Hernandez) (2016)). However, according to other research, loneliness was distinct from social isolation, with the former linked to the absence of an intimate relationship (emotional loneliness) or a wider social network (social loneliness) (de Jong‐Gierveld, van Tilburg, & Dykstra, 2006). Andersson (1998) and Tornstam (1990) asserted that it was reasonable to assume that both levels of loneliness are related and that inconvenience caused by one type can be alleviated by compensation from the other level. The results of the present study cannot shed light on the levels of loneliness experienced by the participants, as the main focus was to assess whether loneliness, as a one‐dimensional concept, influenced HRQoL of older adults with cognitive impairment. However, the different levels of loneliness could be an interesting topic for further research.

The findings from this study are in line with earlier research that investigated HRQoL among older adults (Fagerström & Borglin, 2013; Pan et al., 2015), which emphasizes the importance of purposeful care for this group. Assessing older adults physical and mental health has become more urgent. Mainly due to the increasing proportion of mental illness. Such research is also necessary because older adults with functional impairments, due to physical and cognitive illnesses, can have difficulty meeting basic‐level needs. The latter may affect their ability to find meaning and fulfilment in life which may lead to a poorer mental health. According to Touhy and Jett (2013), mental health is not different in old age, but the level of challenge may be greater. Thus, to support and maintain a good physical and mental health, there is a need for qualitative studies focusing on older adults needs. Such research can be used to develop care based on a person‐centred approach.

### 5.1 Limitations

The present study has some limitation as regards the clinical importance of the results. Dropouts in the health measurement scales (SF‐12 and EQ‐5D) may have affected the results as the participants selected for the analysis in this study was based on the specific MMSE score 20–26 points. A possible explanation for the dropout rate is therefore the measurement of HRQoL was based on self‐reported data, which can lead to an increased risk of loss of information. Further, the validity of self‐report data can be questioned since the respondents all have cognitive impairment. However, as the results of this study showed that HRQoL of the study sample was similar to that of the general Swedish geriatric population, it is reasonable to assume that the internal dropout rate does not alter the clinical importance of the results. Furthermore, the study sample was obtained from a larger population study, where the participants had been randomly selected from the community. Thus, the results of this study may be generalized to a similar population of older adults with cognitive impairment.

Another possible limitation of this study was the use of cut‐off points when analysing the results. When evaluating HRQoL based on health measurement scales, cut‐off points are used to predict a dichotomous outcome. When establishing cut‐off points, although a difference in mortality of 1% may be of little consequence, a difference of 3.1 points on a HRQoL scale is much less comprehensible, as the measurement of health and QoL is subjectively self‐rated. Consequently, there have been attempts to establish minimum standards for clinically important change using cut‐off points (Streiner, 2014). We believe that the cut‐points used in this study do not change the clinical importance of the results.

## 6 CONCLUSION

The results of this study provide an overview of important factors that influence HRQoL of older adults with cognitive impairment. Living at home and having the ability to independently perform ADL without receiving informal care, together with the absence of moderate to severe pain and loneliness, were associated with high HRQoL in the study population. Recognizing the significance of the aforementioned factors is important when adopting a person‐centred approach to support care needs and enhance independence in self‐care activities of older adults with cognitive impairment. It can also increase the possibility of maintaining high HRQoL among older adults with cognitive impairment. The results of this study can be used as a foundation for qualitative research aimed at improving the overall QoL of this group. Further research is needed to explore interventions to support and improve HRQoL among older adults with cognitive impairment.

## CONFLICTS OF INTEREST

The authors declare no conflicts of interest with respect to the research, authorship and/or publication of this article.

## References

[nop2265-bib-0001] Ahn, I. S. , Kim, J.‐H. , Kim, S. , Chung, J. W. , Kim, H. , Kang, H. S. , & Kim, D. K. (2009). Impairment of instrumental activities of daily living in patients with mild cognitive impairment. Psychiatry Investigation, 6, 180–184. 10.4306/pi.2009.6.3.180 20046393 PMC2796066

[nop2265-bib-0002] Altman, D. G. (1991). Practical statistics for medical research. London, UK: Chapman and Hall.

[nop2265-bib-0003] Andersson, L. (1998). Loneliness research and interventions: A review of the literature. Aging and Mental Health, 2, 264–274. 10.1080/13607869856506

[nop2265-bib-0004] Baernholdt, M. , Hinton, I. , Yan, G. , Rose, K. , & Mattos, M. (2012). Factors associated with quality of life in older adults in the United States. Quality of Life Research, 21, 527–534. 10.1007/s11136-011-9954-z 21706127 PMC3593634

[nop2265-bib-0005] Bárrios, H. , Narciso, S. , Guerreiro, M. , Maroco, J. , Logsdon, R. , & de Mendonça, A. (2013). Quality of life in patients with mild cognitive impairment. Aging and Mental Health, 17(3), 287–292. 10.1080/13607863.2012.747083 23215827

[nop2265-bib-0006] Bauermeister, S. , & Bunce, D. (2015). Poorer mental health is associated with cognitive deficits in old age. Aging, Neuropsychology and Cognition, 22, 95–105. 10.1080/13825585.2014.893554 24605782

[nop2265-bib-0007] Beerens, H. C. , Sutcliffe, C. , Renom‐Guiteras, A. , Soto, M. E. , Suhonen, R. , & Zabalegui, A. … RightTimePlaceCare Consortium (2014). Quality of life and quality of care for people with dementia receiving long term institutional care or professional home care: The European RightTimePlaceCare study. Journal of the American Medical Directors Association, 15, 54–61. 10.1016/j.jamda.2013.09.010 24220139

[nop2265-bib-0008] Bircher, J. (2005). Towards a dynamic definition of health and disease. Medicine, Health Care and Philosophy, 8, 335–341. 10.1007/s11019-005-0538-y 16283496

[nop2265-bib-0009] Borglin, G. (2005). *Quality of life among older people. Their experience, need of help, health, social support, everyday activities and sense of coherence* ([PhD thesis]).Lund University: Department of Health Sciences.

[nop2265-bib-0010] Bowling, A. (2001). Measuring disease: A review of disease‐specific quality of life measurement scales (2nd ed.) Buckingham, UK; Phildelphia, PA: Open University Press.

[nop2265-bib-0011] Bowling, A. (2017). Measuring health: A review of subjective health, well‐being and quality of life measurement scales (4th ed.) London, UK: Open University Press.

[nop2265-bib-0012] Comans, T. A. , Nguyen, K.‐H. , Mulhern, B. , Corlis, M. , Li, L. i. , Welch, A. , … Ratcliffe, J. (2018). Developing a dementia‐specific preference‐­based quality of life measure (AD‐5D) in Australia: A valuation study protocol. British Medical Journal Open, 8, e018996 10.1136/bmjopen-2017-018996 PMC5781065 29358437

[nop2265-bib-0013] Cooper, C. , Mukadam, N. , Katona, C. , Lyketsos, C. G. , Ames, D. , & Rabins, P. … World Federation of Biological Psychiatry – Old Age Taskforce (2012). Systematic review of the effectiveness of non‐pharmacological interventions to improve quality of life of people with dementia. International Psychogeriatrics, 24, 856–870. 10.1017/S1041610211002614 22244371

[nop2265-bib-0014] de Jong‐Gierveld, J. , van Tilburg, T. G. , & Dykstra, P. A. (2006). Loneliness and social isolation In Perlman D., & Vangelisti A. (Eds.), The Cambridge handbook of personal relationships (pp. 485–500). Cambridge, UK: Cambridge University Press.

[nop2265-bib-0015] DeFries, E. , McGuire, L. C. , Andresen, E. M. , Brumback, B. A. , & Anderson, L. A. (2009). Caregivers of older adults with cognitive impairment. Preventing Chronic Disease, 6, A46. 19288989 PMC2687852

[nop2265-bib-0016] Diener, E. (2000). Subjective well‐being: The science of happiness and a proposal for a national index. American Psychologist, 55, 34–43. 10.1037/0003-066X.55.1.34 11392863

[nop2265-bib-0017] Dolan, P. (1996). Modelling valuations for health states: The effect of duration. Health Policy, 38, 189–203. 10.1016/0168-8510(96)00853-6 10162421

[nop2265-bib-0018] Fagerström, C. , & Borglin, G. (2013). Mobility, functional ability and health‐related quality of life among people of 60 years or older. Aging Clinical and Experimental Research, 22, 387–394. 10.1007/BF03324941 21422794

[nop2265-bib-0019] Folstein, M. F. , Folstein, S. E. , & McHugh, P. R. (1975). “Mini‐mental state”. A practical method for grading the cognitive state of patients for the clinician. Journal of Psychiatric Research, 12(3), 189–198. 10.1016/0022-3956(75)90026-6 1202204

[nop2265-bib-0020] Franzén, K. , Saveman, B.‐I. , & Blomqvist, K. (2007). Predictors for health related quality of life in persons 65 years or older with chronic heart failure. European Journal of Cardiovascular Nursing, 6, 112–120. 10.1016/j.ejcnurse.2006.06.001 16859996

[nop2265-bib-0021] Gerino, E. , Rollè, L. , Sechi, C. , & Brustia, P. (2017). Loneliness, resilience, mental health and quality of life in old age: A structural equation model. Frontiers in Psychology, 8:2003 10.3389/fpsyg.2017.02003 PMC5694593 29184526

[nop2265-bib-0022] Glisky, E. L. (2007). Changes in cognitive function in human aging In D. R. Riddle(Ed.), Brain aging: Models, methods and mechanisms (pp. 3–20). Boca Raton, FL: CRC Press/Taylor & Francis 21204355

[nop2265-bib-0023] Guyatt, G. H. (1993). Measuring health‐related quality of life. Annals of Internal Medicine, 118, 622 10.7326/0003-4819-118-8-199304150-00009 8452328

[nop2265-bib-0024] Hernandez, L. M. , Blazer, D. G. , & Institute of Medicine (US) Committee on Assessing Interactions Among Social, Behavioral, and Genetic Factors in Health (2006). The impact of social and cultural environment on health. Washington, DC: National Academies Press Retrieved from https://www.ncbi.nlm.nih.gov/books/NBK19924/

[nop2265-bib-0025] Hopman, W. M. , Harrison, M. B. , Coo, H. , Friedberg, E. , Buchanan, M. , & VanDenKerkhof, E. G. (2009). Associations between chronic disease, age and physical and mental health status. Chronic Diseases in Canada, 29, 108–116. 19527569

[nop2265-bib-0026] Hosmer, D. W. , & Lemeshow, S. (2013). Applies logistic regression. New York, NY: Wiley.

[nop2265-bib-0027] Hounsome, N. , Orrell, M. , & Edwards, R. T. (2011). EQ‐5D as a quality of life measure in people with dementia and their carers: Evidence and key issues. Value in Health, 14(2), 390–399. 10.1016/j.jval.2010.08.002 21402307

[nop2265-bib-0028] Huber, M. , Knottnerus, J. A. , Green, L. , Horst, H. V. D. , Jadad, A. R. , Kromhout, D. , … Smid, H. (2011). How should we define health? BMJ, 343, d4163–d4163. 10.1136/bmj.d4163 21791490

[nop2265-bib-0029] Jenkinson, C. , & Layte, R. (1997). Development and testing of the UK SF‐12 (short form health survey). Journal of Health Services Research and Policy, 2(1), 14–18. 10.1177/135581969700200105 10180648

[nop2265-bib-0030] Johansson, M. M. , Marcusson, J. , & Wressle, E. (2012). Cognition, daily living and health‐related quality of life in 85‐year‐olds in Sweden. Neuropsychology, Development and Cognition. Section B, Aging, Neuropsychology and Cognition, 19, 421–432. 10.1080/13825585.2011.629290 22126333

[nop2265-bib-0031] Johansson, M. M. , Marcusson, J. , & Wressle, E. (2015). Cognitive impairment and its consequences in everyday life: Experiences of people with mild cognitive impairment or mild dementia and their relatives. International Psychogeriatrics, 27(6), 949–958. 10.1017/S1041610215000058 25644289

[nop2265-bib-0032] Kang, H.‐W. , Park, M. , & Wallace (Hernandez), J. P. (2016). The impact of perceived social support, loneliness and physical activity on quality of life in South Korean older adults. Journal of Sport and Health Science, 7(2), 237–244. 10.1016/j.jshs.2016.05.003 30356448 PMC6180534

[nop2265-bib-0033] Katz, S. , Ford, A. B. , Moskowitz, R. W. , Jackson, B. A. , & Jaffe, M. W. (1963). Studies of illness in the aged: The index of adl: A standardized measure of biological and psychosocial function. JAMA, 185, 914–919. 10.1001/jama.1963.03060120024016 14044222

[nop2265-bib-0034] Kindig, D. A. , Asada, Y. , & Booske, B. (2008). A population health framework for setting national and state health goals. JAMA, 299, 2081–2083. 10.1001/jama.299.17.2081 18460667

[nop2265-bib-0035] Lagergren, M. , Fratiglioni, L. , Hallberg, I. R. , Berglund, J. , Elmståhl, S. , Hagberg, B. , … Wimo, A. (2004). A longitudinal study integrating population, care and social services data. The Swedish National study on Aging and Care (SNAC). Aging Clinical and Experimental Research, 16, 158–168. 15195992 10.1007/BF03324546

[nop2265-bib-0036] Lawton, M. P. , & Brody, E. M. (1969). Assessment of older people: Self‐maintaining and instrumental activities of daily living. The Gerontologist, 9, 179–186. 10.1093/geront/9.3_Part_1.179 5349366

[nop2265-bib-0037] Leist A., Kulmala J., & Nyqvist F. (Eds.) (2014). Health and cognition in old age. From biomedical and life course factors to policy and practice. Cham, Switzerland: Springer International Publishing.

[nop2265-bib-0038] Leow, M.‐K.‐S. , Griva, K. , Choo, R. , Wee, H.‐L. , Thumboo, J. , Tai, E. S. , & Newman, S. (2013). Determinants of Health‐Related Quality of Life (HRQoL) in the Multiethnic Singapore Population – A National Cohort Study. PLoS ONE, 8, e67138 10.1371/journal.pone.0067138 23826215 PMC3695030

[nop2265-bib-0039] Lethin, C. (2016). *Informal caregivers of older persons with dementia in eight European countries. Experiences, support, well‐being and burden* . ([PhD thesis]). Lund University: Faculty of Medicine.

[nop2265-bib-0040] Loboprabhu, S. , & Molinari, V. (2012). Severe loneliness in community‐dwelling aging adults with mental illness. Journal of Psychiatric Practice, 18, 20–28. 10.1097/01.pra.0000410984.15852.59 22261980

[nop2265-bib-0041] Mlinac, M. E. , & Feng, M. C. (2016). Assessment of activities of daily living, self‐care and independence. Archives of Clinical Neuropsychology, 31, 506–516. 10.1093/arclin/acw049 27475282

[nop2265-bib-0042] Murman, D. L. (2015). The impact of age on cognition. Seminars in Hearing, 36, 111–121. 10.1055/s-0035-1555115 27516712 PMC4906299

[nop2265-bib-0044] Orgeta, V. , Edwards, R. T. , Hounsome, B. , Orrell, M. , & Woods, B. (2015). The use of the EQ‐5D as a measure of health‐related quality of life in people with dementia and their carers. Quality of Life Research, 24, 315–324. 10.1007/s11136-014-0770-0 25129054 PMC4317511

[nop2265-bib-0045] Pan, C.‐W. , Wang, X. , Ma, Q. , Sun, H.‐P. , Xu, Y. , & Wang, P. (2015). Cognitive dysfunction and health‐related quality of life among older Chinese. Scientific Reports, 5, 17301 10.1038/srep17301 26601612 PMC4658548

[nop2265-bib-0046] Prince, M. , Wimo, A. , Guerchet, M. , Ali, G. , Wu, Y. , & Prina, A. (2015). *World Alzheimer Report 2015: The global impact of dementia: an analysis of prevalence, incidence, cost and trends* .

[nop2265-bib-0047] Pusswald, G. , Tropper, E. , Kryspin‐Exner, I. , Moser, D. , Klug, S. , Auff, E. , … Lehrner, J. (2015). Health‐related quality of life in patients with subjective cognitive decline and mild cognitive impairment and its relation to activities of daily living. Journal of Alzheimer's Disease, 47, 479–486. 10.3233/JAD-150284 26401569

[nop2265-bib-0048] Qiu, C. , von Strauss, E. , Bäckman, L. , Winblad, B. , & Fratiglioni, L. (2013). Twenty‐year changes in dementia occurrence suggest decreasing incidence in central Stockholm, Sweden. Neurology, 80, 1888–1894. 10.1212/WNL.0b013e318292a2f9 23596063

[nop2265-bib-0049] Reppermund, S. , Brodaty, H. , Crawford, J. D. , Kochan, N. A. , Draper, B. , Slavin, M. J. , … Sachdev, P. S. (2013). Impairment in instrumental activities of daily living with high cognitive demand is an early marker of mild cognitive impairment: The Sydney Memory and Ageing Study. Psychological Medicine, 43, 2437–2445. 10.1017/S003329171200308X 23308393

[nop2265-bib-0050] Saracci, R. (1997). The World Health Organization needs to reconsider its definition of health. BMJ, 314, 1409–1410. 9161320 10.1136/bmj.314.7091.1409 PMC2126653

[nop2265-bib-0051] Schulz, R. , & Martire, L. M. (2004). Family caregiving of persons with dementia: Prevalence, health effects and support strategies. The American Journal of Geriatric Psychiatry, 12, 240–249. 10.1097/00019442-200405000-00002 15126224

[nop2265-bib-0052] Singh, R. , & Dixit, S. (2010). Health‐related quality of life and health management. Journal of Health Management, 12(2), 153–172. 10.1177/097206341001200204

[nop2265-bib-0054] Streiner, D. L. (2014). Health measurement scales. A practical guide to their development and use (5th ed.) Oxford, UK: Open University Press.

[nop2265-bib-0055] Sullivan, M. , Karlsson, J. , & Taft, C. (1997). *Health questionnaire: Swedish Manual* . Göteborg, Sweden: Sektionen för vårdforskning, Universitetet MFG.

[nop2265-bib-0056] Teeters, D. A. , Moua, T. , Li, G. , Kashyap, R. , Biehl, M. , Kaur, R. , … Caples, S. M. (2016). Mild cognitive impairment and risk of critical illness. Critical Care Medicine, 44, 2045–2051. 10.1097/CCM.0000000000001842 27441907 PMC5069082

[nop2265-bib-0057] Tornstam, L. (1990). Dimensions of loneliness. Aging, 2, 259–265. 10.1007/BF03323926 2094364

[nop2265-bib-0058] Touhy, T. A. , & Jett, K. F. (2013). Ebersole & Hess' toward healthy aging ‐ E‐Book: Human needs and nursing response. London, UK: Elsevier Health Sciences.

[nop2265-bib-0059] Ware, J. E. (1987). Standards for validating health measures: Definition and content. Journal of Chronic Diseases, 40, 473–480. 10.1016/0021-9681(87)90003-8 3298292

[nop2265-bib-0060] Ware, J. , Kosinski, M. , & Keller, S. D. (1996). A 12‐item short‐form health survey: Construction of scales and preliminary tests of reliability and validity. Medical Care, 34, 220–233. 10.1097/00005650-199603000-00003 8628042

[nop2265-bib-0061] Wenger, G. C. , Davies, R. , Shahtahmasebi, S. , & Scott, A. (1996). Social isolation and loneliness in old age: Review and model refinement. Ageing and Society, 16, 333–358. 10.1017/S0144686X00003457

[nop2265-bib-0062] Wilkes, R. (1978). ´General philosphy and attitudes to aging´, 9, 14–16. 10.1007/BF03323926.

[nop2265-bib-0063] Wilson, N. , Hilmer, S. , March, L. , Cameron, I. , Lord, S. , Mason, R. , & Sambrook, P. (2011). Physical functioning measures and risk of falling in older people living in residential aged care facilities. Therapeutic Advances in Musculoskeletal Disease, 3, 9–15. 10.1177/1759720X10389848 22870462 PMC3383537

[nop2265-bib-0064] Winblad, B. , Amouyel, P. , Andrieu, S. , Ballard, C. , Brayne, C. , Brodaty, H. , … Zetterberg, H. (2016). Defeating Alzheimer's disease and other dementias: A priority for European science and society. The Lancet Neurology, 15(5), 455–532. 10.1016/S1474-4422(16)00062-4 26987701

[nop2265-bib-0065] WMA (2013). *WMA Declaration of Helsinki ‐ Ethical principles for medical research involving human subjects* . Retrieved from http://www.wma.net/en/30publications/10policies/b3/ Accessed April 10, 2018

[nop2265-bib-0066] World Health Organization (1948). *Preamble to the Constitution of WHO as adopted by the International Health Conference* , New York, 19 June ‐ 22 July 1946; singed on 22 July by the representatives of 61 States (Official Records of the World Health Organization, no 2, p. 100) and entered into force on 7 April 1948.

